# Schwannoma Gene Therapy via Adeno-Associated Viral Vector Delivery of Apoptosis-Associated Speck-like Protein Containing CARD (ASC): Preclinical Efficacy and Safety

**DOI:** 10.3390/ijms23020819

**Published:** 2022-01-13

**Authors:** Sherif G. Ahmed, Casey A. Maguire, Shiliang Alice Cao, Gary J. Brenner

**Affiliations:** 1 Department of Anesthesiology, Critical Care, Pain Medicine, Massachusetts General Hospital, Harvard Medical School, Boston, MA 02129, USA; Sherif.Ahmed@MGH.Harvard.edu (S.G.A.); scao1@mgh.harvard.edu (S.A.C.); 2 Department of Neurology, Massachusetts General Hospital, Harvard Medical School, Boston, MA 02114, USA; cmaguire@mgh.harvard.edu

**Keywords:** schwannoma, ASC, gene therapy, AAV, neutralizing antibodies

## Abstract

Schwannomas are tumors derived from Schwann-lineage cells, cells that protect and support myelinated nerves in the peripheral nervous system. They are typically slow-growing, encapsulated and benign. These tumors develop along peripheral, spinal and cranial nerves causing pain, sensory-motor dysfunction and death. Primary treatment for schwannoma is operative resection which can be associated with significant morbidity. Pharmacotherapy is largely restricted to bevacizumab, which has minimal or no efficacy for many patients and can be associated with treatment-limiting adverse effects. Given the suffering and morbidity associated with schwannoma and the paucity of therapeutic options, there is an urgent need for safe and effective therapies for schwannomas. We previously demonstrated that adeno-associated virus serotype 1 (AAV1) vector mediated delivery of the inflammasome adaptor protein, apoptosis-associated speck-like protein containing a caspase recruitment domain (*ASC*) under the control of the P0 promoter, produced a prolonged reduction in tumor volume and tumor-associated pain in human xenograft and mouse syngeneic schwannoma models. Here, we present data essential for the translation of our AAV1-P0-ASC schwannoma gene therapy to clinical trials. We determine the minimum effective dose of AAV1-P0-hASC required to induce an anti-tumor effect in the xenograft human-schwannoma model. We also show that the presence of preexisting AAV1 immunity does not alter the antitumor efficacy of AAV-P0-mASC in a syngeneic mouse schwannoma model. Furthermore, the maximum deliverable intratumoral dose of AAV1-P0-ASC was not associated with neuronal toxicity in immunocompetent mice. Taken together, these safety and efficacy data support the translation of the AAV1-P0-ASC schwannoma gene therapy strategy to clinical trials.

## 1. Introduction

Schwannomas are slow-growing tumors derived from Schwann-lineage cells [1,2]. Depending on location and size, these tumors can cause a variety of gain- and loss-of-function neurological pathologies including deafness, dizziness, pain, motor dysfunction and even death [3,4]. Schwannomas may arise sporadically or as part of the debilitating genetic syndromes neurofibromatosis type 2 (NF2) and schwannomatosis [5]. Current treatment of schwannomas is largely limited to surgical resection, which can be associated with significant morbidity including deafness and facial paralysis due to cranial nerve VIII and VII damage, respectively, as well as sensory and motor dysfunction following injury to peripheral nerves [6], and is not always possible due to the risk of peripheral nerve, spinal cord, or brainstem damage [7]. Schwannomas frequently present in multiple locations with new tumors arising throughout an affected individual’s life, thus further limiting the utility of surgical resection [3]. There are no FDA-approved drugs for NF2 disease [8] and the only well-accepted pharmacotherapeutic is the angiogenesis inhibitor, bevacizumab (Avastin), which has limited value for many patients due to either lack of efficacy or side effects [9,10,11]. The debilitation and suffering endured by individuals with schwannoma (as well as their families), in combination with the paucity of therapeutic options, makes the treatment of schwannoma a major unmet medical need.

Gene therapy for schwannoma via direct intratumoral (I.T.) injection is an attractive treatment strategy due to the slow growth of the tumor and the ability to readily localize the tumor using ultrasound and/or magnetic resonance imaging (MRI), respectively. A major advantage of gene therapy over surgical resection is that the former utilizes a substantially less invasive procedure (image guided injection). Our published data demonstrate that I.T. injection of an adeno-associated virus serotype 1 vector (AAV1) encoding proapoptotic transgenes including as caspase-1 (*ICE*) [12], N terminal fragment of gasdermin-d [13] or apoptosis-associated speck-like protein containing a CARD (caspase recruitment domain), known as *ASC or PYCARD* [14] in each case under control of rP0 (r = rat) cells resulted in significant reduction in tumor growth in human-derived schwannoma xenograft mouse models [12]. In these and other studies, we demonstrated that rP0 is optimal for both tissue specificity, and critically, protecting neuronal tissue from potential off-target toxicity [12,14,15,16].

In contradistinction to caspase-1 and gasdermin-d, ASC is an adaptor protein that activates multiples apoptotic and pyroptotic factors by mediating the assembly of large signaling complexes in apoptotic, inflammatory and non-inflammatory pathways [17,18]. ASC, via its CARD site, promotes apoptosis by binding to and activating multiple intracellular enzymes including caspase-1, caspase-8 and caspase-9 [18,19]. Additionally, *ASC*, which is also referred to as target of methylation-induced silencing (*TMS1*), was reported to be aberrantly methylated and silenced in multiple different cancers [20,21]. We have previously shown that ASC is specifically suppressed through methylation in both NF2-associated and sporadic schwannomas, suggesting a possible regulatory role for ASC in controlling the proliferation of these tumors [14]. Using our method of directly injecting our gene therapy vector into schwannomas via AAV1 vector under control of rP0, we found that the apoptotic death of schwannoma cells occurred without toxicity to other cells comprising the associated peripheral nerve. AAVs are naturally occurring in the environment and while non-pathogenic in humans there is a concern that efficacy of AAV-based gene therapies may be limited by the presence of AAV neutralizing antibodies. Indeed, in some clinical trials the pre-existing AAV immunity has affected efficacy and safety of intravenously delivered AAVs [22]. The prevalence of AAV1 neutralizing antibodies in the human population varies regionally and has been reported to be between 20% to 59% [23,24,25].

In the present work, we further evaluate our AAV1-rP0-ASC schwannoma gene therapy as a bridge to clinical trials. Data presented include establishing minimum effective dose (MED), rigorous assessment of toxicity, and evaluation of pre-existing AAV1 immunity on I.T. AAV1-rP0-ASC efficacy and safety. 

## 2. Results

### 2.1. Determination of the Minimum Effective Dose of AAV1-rP0-hASC

**Figure 1:**
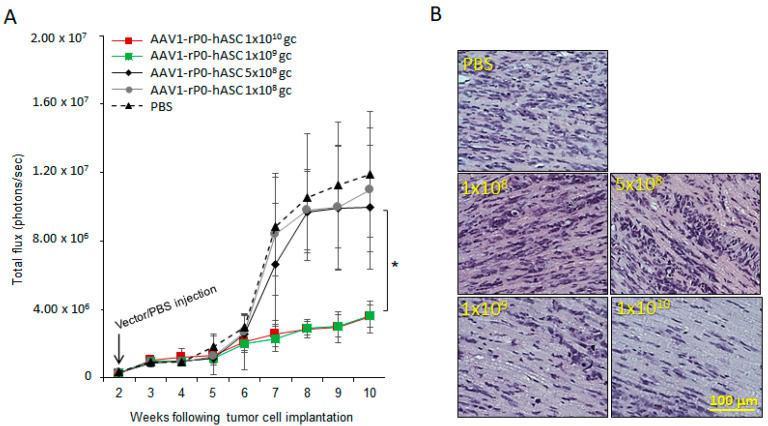
Determination of the minimum effective dose (MED) of AAV1-rP0-hASC control of HEI-193 intrasciatic schwannomas. (**A**) Growth kinetics of intrasciatic HEI-193 xenograft schwannomas following intra-tumoral (I.T.) with different doses of AAV1-rP0-hASC or PBS control (*n* = 8 per group). ANOVA indicated a significant effect of 1 × 10^10^ gc and 1 × 10^9^ gc AAV1-rP0-hASC compared to PBS, but not of 5 × 10^8^ gc or 1 × 10^8^ gc AAV1-rP0-hASC (* *p* < 0.05). (**B**) Hematoxylin and eosin (H&E) staining demonstrated abundant, mitotically active hematoxylin-positive HEI-193 tumor cells in the mice which experimental schwannomas were injected I.T. with 5 × 10^8^ gc and 1 × 10^8^ gc of AAV1-rP0-hASC or PBS, but fewer tumor cells were in tumor-bearing nerves injected with 1 × 10^10^ gc and 1 × 10^9^ gc AAV1-rP0-hASC.

We determined the minimal effective does (MED) of AAV1-rP0-hASC using our published xenograft human-schwannoma model [26]. AAV1-rP0-hASC or phosphate-buffered saline (PBS) vehicle was injected I.T. two-weeks following intrasciatic cellular implantation of the HEI-193FC human schwannoma cell line and tumor development followed via bioluminescent imaging. There was significant control of tumor growth in the mice injected with either 1 × 10^10^ gc or 1 × 10^9^ gc AAV1-rP0-hASC, compared to the PBS injected tumors (Figure 1A). Lower AAV1-rP0-hASC doses (5 × 10^8^ gc and 1 × 10^8^ gc) tested did not result in control of tumor growth. These data were further validated by hematoxylin and eosin (H&E) staining of tumor-bearing nerves at the time of sacrifice. Mice injected with 1 × 10^10^ gc or 1 × 10^9^ gc AAV1-rP0-hASC showed a noticeable reduction in H&E positive tumor cells, compared to 5 × 10^8^ gc, 1 × 10^8^ gc or PBS injected mice, which showed abundant H&E positive tumor cells in the injected nerves (Figure 1B). Thus, MED was determined to be 1 × 10^9^ gc.

### 2.2. The Maximum Intrasciatic Injectable Dose of AAV1-rP0-mASC Is Not Associated with Neurotoxicity in Naïve Immunocompetent Mice

**Figure 2:**
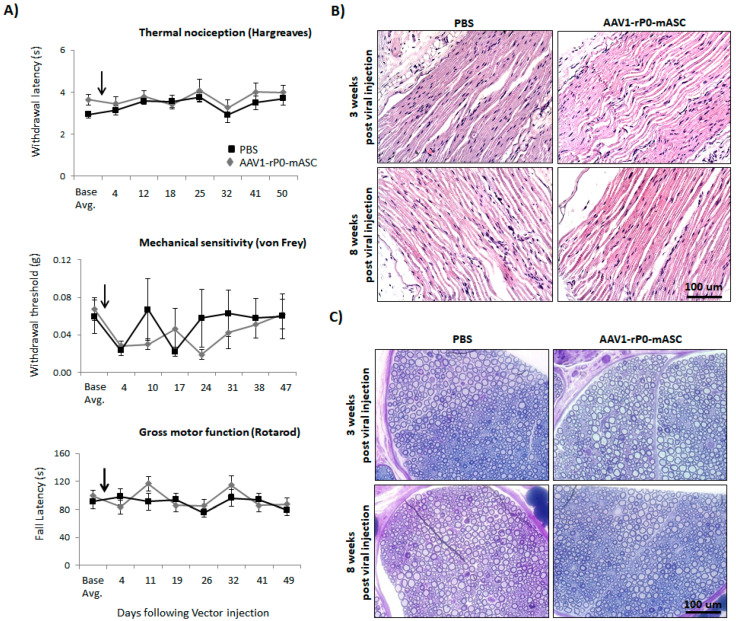
AAV1-rP0-mASC intra-sciatic injection of naïve immunocompetent C57BL/6 mice has no effect on sensory or gross motor function in vivo. (**A**) Effects of intrasciatic AAV1-rP0-mASC or PBS injection on pain behaviors, both thermal (Hargreaves test, **top**) and mechanical sensitivity (von Frey method, **middle**), and on gross motor function (rotarod, **bottom**) were evaluated at 2- to 7-day intervals for approximately 7-weeks following vector/PBS injection (arrows show time of intrasciatic injection). Repeated measures ANOVA was used to (1) compare behaviors of the AAV1-rP0-mASC and PBS injected mice, as well as (2) to conduct within group comparisons post virus injection at each time point to their respective baseline value. Results are represented as the mean ± SEM; *n* = 8 mice per group. (**B**,**C**) Neuropathological evaluation of sciatic nerve after AAV1-rP0-mASC or PBS injection; (**B**) hematoxylin and eosin and (**C**) toluidine blue staining of myelin in Epon-embedded sciatic nerve of naïve C57BL/6 mice. Cross sections of sciatic nerve collected 3- and 8-weeks post virus injection demonstrates essentially normal myelinated nerves with rare axonal degeneration.

We then evaluated AAV1-rP0-mASC neuronal toxicity following vector injection in the sciatic nerve of immune competent mice. We compared the maximum injectable dose of AAV1-rP0-mASC (1 × 10^10^ gc delivered in 2 uL PBS) with PBS (2 uL) following intrasciatic injection of non-tumor-bearing naïve C57Bl/6 mice. We utilized two behavioral tests to assess pain: Hargreaves’ method and von Frey filaments (Figure 2A). Thermal sensitivity was assessed using the Hargreaves’ method, in which a movable infrared heat source is positioned underneath the hind paw ipsilateral to tumor and withdrawal latency is measured [27]. Mechanical sensitivity (allodynia) was assessed using von Frey filaments to establish withdrawal threshold of the hind paw ipsilateral to the AAV1-rP0-mICE injected sciatic nerve [28]. Gross motor performance, as further behavioral measure of neurotoxicity, was assessed by the accelerating rotarod test [29]. Thermal sensitivity (Hargreaves) and mechanical sensitivity (Von Frey) did not differ between AAV1-rP0-mASC and PBS injected mice over 5 weeks after injections, and for all groups the Hargreaves withdrawal latency and von Frey threshold values returned to pre-injection baseline (i.e., normalization of pain sensitivity) in these mice (Figure 2A). Thus, there was no sustained change in sensory function associated with AAV1-rP0-mASC per se, though there was a transient change in sensory thresholds to the injection procedure. Gross motor function (rotarod) was not affected by AAV1-rP0-mASC or PBS injection (Figure 2A). In the same experiment, we histologically evaluated via H&E and Epon staining AAV1-rP0-mASC and PBS injected sciatic nerves at 3 and 8 weeks post-injection. H&E staining showed essentially normal nerves with rare axonal degeneration, normal myelination and axonal integrity in the mice injected with either vector (Figure 2B). Epon embedded sections confirmed the H&E results demonstrating that normal density of small myelinated and large myelinated fibers (Figure 2C). There was no significant inflammation in either AAV1-rP0-mASC or PBS injected nerves. Blood vessels were unremarkable and did not differ between treatments. There were rare degenerating axons in the PBS-injected nerves and scant macrophages in AAV-rP0-mASC injected nerves. These findings most likely represent response to trauma from injection itself as they are not seen consistently across animals and sections.

### 2.3. Preexisting AAV1 Immunity Does Not Alter Anti-Tumor Efficacy or Safety of I.T. AAV1-rP0-mASC 

**Figure 3:**
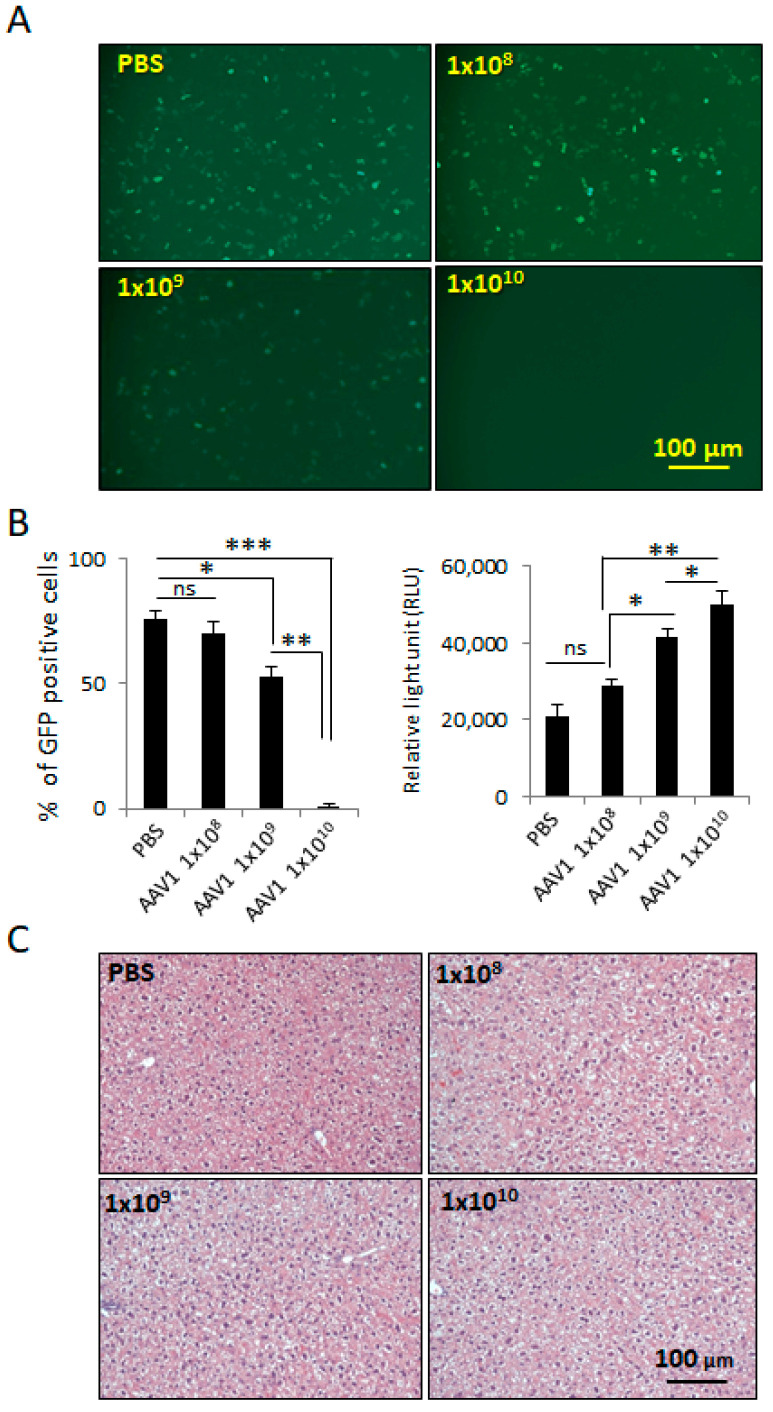
Induction of anti-AAV1-antibodies in C57BL/6 mice injected I.P. with different doses of AAV1-rP0-null. (**A**) Collected blood sera from mice 1-week following AAV1-rP0-null 1 × 10^10^ gc injection resulted in complete AAV1-CBA-GFP neutralization as shown in reduced GFP expression when added to HeLa cells. (**B**) Quantification of the GFP positive cells showed that 1 × 10^10^ gc of AAV1-rP0-null injection showed the lowest percentage of GFP positive cells (**Left**). ELISA assay of AAV1 antibodies showed that 1 × 10^10^ gc of AAV1-rP0-null injection produced the highest titer of anti-AAV1-antibodies (**right**). (**C**) Liver H&E staining of the AAV1-immunized mice did not show inflammatory changes, compared to PBS injected mice. One-way ANOVA was utilized for statistical analysis. Results are represented as the mean ± SEM; *n* = 3 mice per group. * *p* < 0.05, ** *p* < 0.01, *** *p* < 0.001.

We next investigated the effect of AAV1 immunity on efficacy and safety of AAV1-rP0-mASC. We first determined the minimum AAV1 vector dose required to induce AAV1-neutralizing antibodies. Immunocompetent naïve C57Bl/6 mice were I.P. injected with different doses of a control vector lacking a transgene sequence downstream of rP0 (AAV1-rP0-null). One week after injection of the vector, the injected mice were harvested, and their blood sera evaluated for presence of AAV1 antibodies. The presence of AAV1 antibodies in the sera was confirmed using neutralization assay in HeLa cells [30] and ELISA assay [31]. For the neutralization assay, AAV1-CBA-GFP vectors were mixed with dilutions of blood sera from the mice injected with different doses of AAV1-rP0-null or PBS (*n* = 3/group) and incubated for 1 h at 37 °C before adding the mixture to HeLa cells for 1 h at 37 °C. Media were placed on the cells and the cells were incubated for 48 h before quantification with GFP expression, which was normalized to the transduction of the vector in the absence of serum. Our data showed that sera from mice injected with AAV1-rP0-null 1 × 10^10^ gc induced complete AAV neutralization as revealed by lack of GFP expression in the transduced HeLa cells (Figure 3A,B). We further confirmed the presence of AAV1 antibodies in the sera of the injected mice by ELISA assay, which showed that 1 × 10^10^ gc of AAV1-rP0-null induced the most significant antibodies, compared to the PBS injected mice (Figure 3B). The AAV1 injected mice did not show any signs of liver toxicity as revealed by H & E staining, compared to the PBS controls (Figure 3C).

**Figure 4:**
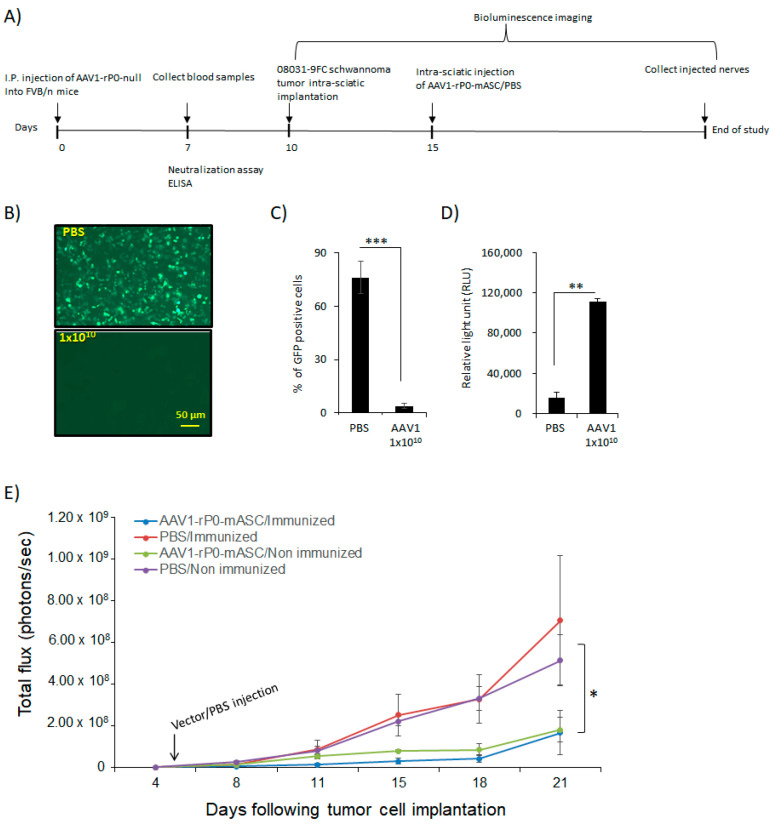
AAV1-rP0-mASC intra-tumoral injection of AAV1-preimmunized immunocompetent mouse schwannoma model slows tumor growth. (**A**) schematic diagram for the experimental design of the effect of AAV1 pre-existing immunity on the efficacy of the intratumoral AAV1-rP0-mASC injection into a syngeneic mouse schwannoma model in immune competent mice. (**B**) AAV1-rP0-null (1 × 10^10^ gc) or PBS were injected I.P. into immunocompetent FVB/*n* mice while blood sera were collected 1-week post injection. Dilutions of the collected sera were mixed with AAV1-CBA-GFP. After incubation for 1 h, mixtures were added to HeLa cells and two days later GFP expression were monitored. (**C**) Quantification of the GFP positive cells showed that AAV1-rP0-null injection resulted in complete neutralization of AAV1-CBA-GFP and thus reduced GFP expression compared to the sera collected from mice injected with PBS. (**D**) ELISA assay of AAV1 antibodies showed AAV1-rP0-null injection produced more significant AAV1 antibodies, compared to PBS. One-way ANOVA was utilized for statistical analysis. Results are represented as the mean ± SEM; *n* = 3 mice per group. ** *p* < 0.01, *** *p* < 0.001. (**E**) Intra-tumoral injection of the implanted syngeneic mice 5 days post-implantation of mouse 08031-9FC cells with AAV1-rP0-mASC vs. PBS control (*n* = 8 per group) in both AAV1 preimmunized and naïve mice. A significant difference was shown in the tumor growth of mice injected with AAV1-rP0-mASC in naïve and preimmunized mice, compared to the PBS injected groups. Repeated measures ANOVA was used to compare the tumor growth difference between the 4 groups. Results are represented as the mean ± SEM; (* *p* < 0.05).

We then analyzed AAV1-rP0-mASC in vivo antitumor efficacy in AAV1-immunized FVB/*n* mouse-schwannoma-bearing mice [13]. We generated and immunized our published syngeneic murine schwannoma model via implanting mouse-08031-9 schwannoma cells in which we expressed firefly luciferase (FC) in the sciatic nerve of immunocompetent syngeneic FVB/*n* mice. 10 days prior to implantation of tumor cells, we injected mice I.P. with 1 × 10^10^ gc of AAV1-rP0-null to generate AAV1-neutralizing antibodies. Sera were collected 1-week post vector injection and the presence of anti-AAV1-immunoglubulins was confirmed by neutralization and ELISA assays (Figure 4B–D). Mouse 08031-9-FC cell were implanted in the sciatic nerve three days following sera collection, and tumor growth was monitored non-invasively using bioluminescence imaging. Tumor-bearing sciatic nerves were injected with AAV1-rP0-mASC or PBS 5 days post schwannoma cell implantation. The injected mice were divided into four groups: (1) AAV1-rP0-mASC I.T. injection in naïve (non-immunized mice), (2) AAV1-rP0-mASC I.T. injection in AAV1-immunized mice, (3) PBS I.T. injection in naïve (non-immunized mice) and (4) PBS I.T. injection in AAV1-immunized mice. Our data demonstrate that the intratumoral injection of AAV1-rP0-mASC led to a significant reduction in the growth of mouse schwannoma syngeneic tumors in both non immunized and AAV1-immunized mice, compared with PBS control groups as revealed by repeated measures ANOVA, and critically, there was no difference in AAV1-rP0-mASC mediated schwannoma control between AAV1 immune and non-immune mice (Figure 4E).

**Figure 5:**
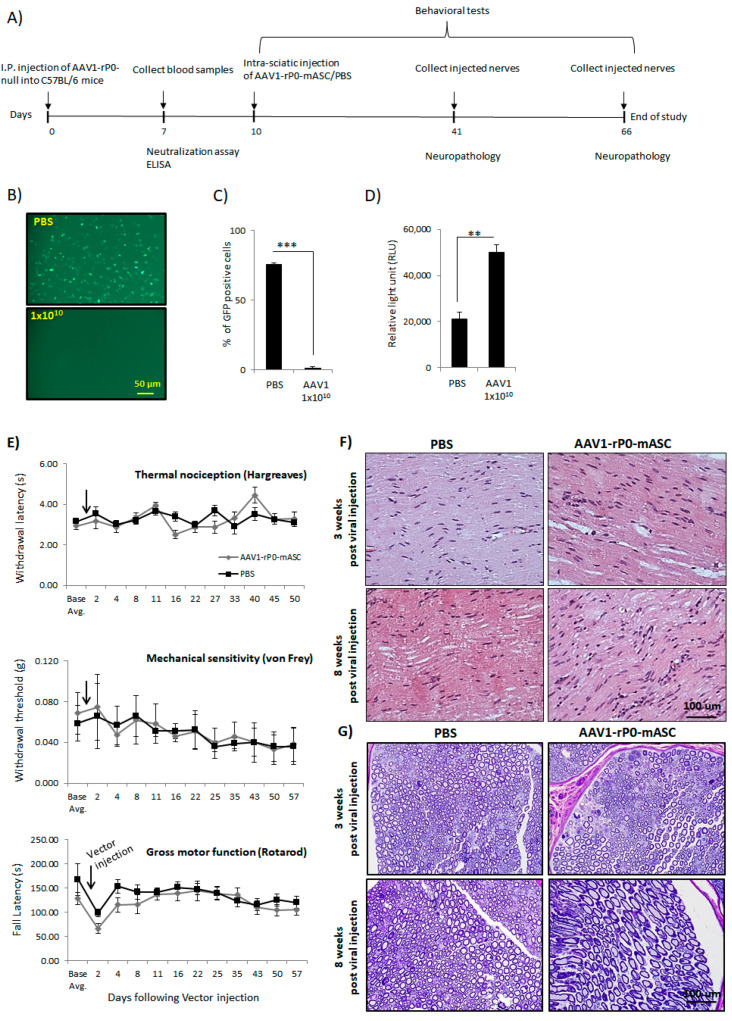
Preexisting AAV1 immunity does not affect safety of intrasciatic AAV1-rP0-mASC in C57BL/6 mice. (**A**) Schematic diagram for the experimental design. (**B**) AAV-rP0-null (1 × 10^10^ gc) or PBS was injected I.P. into immunocompetent C57BL/6 mice and blood sera were collected 1 week post injection. Dilutions of the collected sera were mixed with AAV1-CBA-GFP, added to HeLa cells, and two days later GFP expression were monitored. (**C**) Quantification of the GFP positive cells showed that AAV1-rP0-null injection resulted in reduced GFP expression compared to the sera collected from PBS-injected mice. (**D**) ELISA for AAV1 antibodies showed AAV1-rP0-null injected mice had more anti-AAV1-antibody than PBS-injected animals. One-way ANOVA was utilized for statistical analysis. Results are represented as the mean ± SEM; *n* = 3 mice per group. ** *p* < 0.01, *** *p* < 0.001. (**E**) Effect of intrasciatic AAV1-rP0-mASC or PBS injection on pain behaviors, thermal nociception (Hargreaves test, **top**) and mechanical sensitivity (von Frey method, **middle**), and on gross motor function (rotarod, **bottom**) was evaluated at 2- to 7-day intervals for approximately 7 weeks following vector/PBS injection. Arrows show time of vector/PBS injection. Repeated measures ANOVA was used to compare behaviors of the AAV1-rP0-mASC and PBS injected mice, as well as within each group comparison post injection at each time point to their respective baseline value. Results are represented as the mean ± SEM; *n* = 8 mice per group. (**F**,**G**) Neuropathological evaluation of sciatic nerves following AAV1-rP0-mASC or PBS injection in AAV1 immune C57BL/6 mice. (**F**) Hematoxylin and eosin (**G**) and toluidine blue staining of myelin in Epon-embedded sciatic nerves. Cross sections of nerves collected 3- and 8-weeks post AAV1-rP0-mASC injection shows essentially normal myelinated nerves with rare axonal degeneration, and no difference from the PBS injected nerves.

In a separate experiment, we investigated the effect of AAV1 neutralizing antibodies on the safety of intra-sciatic injection of AAV1-rP0-mASC in naïve immunocompetent C57Bl/6 mice. C57Bl/6 mice were AAV1-immunized via I.P. injection of 1 × 10^10^ gc of AAV1-rP0-null. One-week post immunization, sera were collected, and the presence of anti-AAV1-antibodies was confirmed by neutralization and ELISA assays (Figure 5B–D). Baseline behavioral measures for sensory and motor functions were measured. The maximum injectable dose (1 × 10^10^ gc injected in 2 uL PBS) of AAV1-rP0-mASC or PBS was injected intrasciatically 10 days post AAV1 immunization. Thermal sensitivity (Hargreaves), mechanical sensitivity (von Frey), and gross motor function (rotarod) assessments were performed throughout the course of the experiment. We did not observe any significant difference in sensory (including pain sensitization) or motor function between the AAV1-rP0-mASC and PBS groups over 5 weeks after injections (Figure 5E). Neuropathological evaluation of AAV1-rP0-mASC and PBS injected nerves of AAV1-immune C57Bl/6 mice was performed to further assess the potential effect of AAV1 immunity on AAV1-rP0-mICE safety. Sciatic nerves were collected at 3- and 8-weeks post AAV1-rP0-mASC/PBS injection. H&E staining showed grossly normal nerves with rare axonal degeneration, normal myelination and axonal integrity; there was no apparent difference between AAV1-rP0-mASC and PBS injected nerves at either time point (Figure 5F). Epon-embedded cross sections also showed that there were no apparent differences between the AAV1-rP0-mASC and PBS injected nerves at either time point; there was minimal inflammation in both groups (Figure 5G). Sciatic nerve-associated blood vessels were unremarkable. There were rare degenerating axons in the and a few macrophages were present in both AAV1-rP0-mASC and PBS injected nerves. These findings most likely represent a response to the physical trauma of injection as they were not seen consistently in all sections.

## 3. Discussion

Treatment of schwannoma represents a major unmet medical need. There is no truly efficacious drug therapy, and the main therapy of schwannoma remains operative resection. However, surgical resection is associated with a significant risk of damage to cranial nerves, peripheral nerves, the spinal cord and the brainstem [32], and in some cases, due to tumor location or number, resection is not feasible. Clinical trials evaluating cancer chemotherapeutics, (including. bevacizumab), and small molecule drugs are ongoing, but the efficacy of the tested drugs has at best been limited, transient and associated with substantial morbidity, including renal failure [7,9]. In this work, we conducted preclinical translational studies to support bridging of one of our published AAV1-P0-ASC schwannoma gene therapy strategies to clinical trials.

Our published studies showed that AAV1 delivering human or mouse *ASC* under control of the rP0 promoter led to control of schwannoma tumor growth in both human-schwannoma xenograft (in nude mice) and mouse schwannoma syngeneic (in immunocompetent mice) models [14]. Evaluation of the efficacy (including determination of minimum effective dose) of AAV1-P0-ASC in preclinical schwannoma models is a crucial step towards translation of this product to clinical trials. We established MED in the human xenograft schwannoma mouse model. In all our previous studies, we used the maximum dose that can be injected directly into tumors, which is 1 × 10^10^ gc injected in 2 uL PBS. This dose was shown to resolve tumor associated pain and control tumor growth in HEI-193 human schwannomas established in nude mice. We have expanded these results by showing a MED of 1 × 10^9^ gc. 

Evaluation of AAV1-P0-ASC associated toxicity is another crucial step towards implementing phase I clinical trials. ASC is a bipartite protein that promotes apoptosis. It acts as an upstream factor of multiple apoptotic pathways [33] and activates multiple caspases through cleavage, such as caspase-8 and caspase-9 [19]. Moreover, ASC activates the known tumor suppressor, proapoptotic p53, upregulates BAX and BID, and suppresses the activity of the survival protein Bcl-2 in human breast cancer cell lines and leukemia [34,35]. The maximum injectable dose of the AAV1-P0-ASC is 1 × 10^10^ gc in 2 uL PBS given the titers of the virus preparations and the limited volume (2 uL) that can be injected into a mouse sciatic nerve. We have built on our published date demonstrating that this dose is not associated with toxicity by testing AAV1-P0-ASC safety and efficacy in the presence of preexisting AAV1 immunity. The prevalence of AAV1 neutralizing antibodies in the human population is 20–59% [23,24,25], and some clinical trials utilizing intravenous injection of AAV have demonstrated vector-induced adverse effects in patients with existing AAV immunity [22], and AAV neutralizing antibodies can effect efficacy [25]. Our therapeutic model involves direct intra-tumoral injection of AAV1 vector into schwannomas developing in the mouse sciatic nerve. Naturally, effects of neutralizing antibodies may differ based on route of vector administration, e.g., intravenous vs. intratumoral. Interestingly, we found that preexisting AAV1 immunity had no effect on either efficacy or safety of AAV1-P0-ASC. This is likely due to two main factors. First, the level of antibodies in peripheral tissue is likely much lower than in blood. Second, the high local concentration of AAV capsids with I.T. injection may allow “dosing through” the antibodies in the tumor microenvironment. This may allow the possibility of redosing of vector if the Schwannoma begins to regrow as AAV vector genomes may be diluted over time. Regarding toxicity, neuronal protection from AAV-P0-ASC is generated in by using the Schwann-lineage cell specific promoter, P0; previous work from our lab demonstrated that there is essentially no expression of P0-controlled transgenes in sensory neurons [12,36]. Neural protection may be further afforded by the fact that ASC is abundantly expressed in peripheral nerves, and thus any leakiness of the P0 promoter and subsequent ASC expression will be negligible in the context of endogenous ASC. 

Overall, this preclinical work further supports AAV1-P0-ASC as promising schwannoma therapy, and represent critical data for the translation of this schwannoma gene therapy strategy to clinical trials. We have found AAV1-P0-ASC to be effective in controlling and limiting the growth of schwannoma tumors in mouse models of human schwannoma. AAV1-P0-ASC would allow for control of tumor size, and the potential for schwannoma debulking in patients via a procedure—image-guided tumor injection—that is less invasive and potentially safer than operative resection. While we have not tested any combination treatments in this work, it is plausible that this gene therapy vector may be combined with currently available treatments to achieve additive or synergistic therapeutic effects, and potentially even result in a reduction of tumor size. The mechanism of action of AAV1-P0-ASC is completely different from those of other schwannoma therapeutics currently being tested; thus, it should not interfere with any current or potential schwannoma treatments. Translation of this AAV1-P0-ASC schwannoma gene therapy to clinical application holds the promise of meeting a major unmet clinical need for patients suffering from schwannoma, particularly for those in whom surgical resection holds high risk or is not an option.

## 4. Materials and Methods

### 4.1. Animals

All animal experiments were approved by and conducted under the oversight of the Massachusetts General Hospital (MGH, Boston, MA, USA) Institutional Animal Care and Use Committee (IACUC protocol number 2014N000211). Five- to seven-week-old male mice; nu/nu, FVB/*n* and C57BL/6 mice (Charles River Laboratories, Wilmington, MA, USA) were kept on a 12:12 light-to-dark cycle with ad libitum access to food, water, and daily health checks by Center for Comparative Medicine staff at MGH.

### 4.2. Cell Culture 

The HEI-193 human schwannoma cell line (from D.J. Lim, House Ear Institute, Los Angeles, CA, USA) was established from a NF2-schwannoma patient, immortalized with human papillomavirus E6/E7 genes and grown as described [37]. The Mouse 08031-9 schwannoma cells (from Dr. Marco Giovannini, Univ. of California, Las Angeles, CA, USA) were grown as described [38]. All cells were infected with lentivirus encoding Fluc and mCherry for bioluminescence imaging and immunohistochemistry, respectively [12]. HeLa cells were cultured as monolayers in Dulbecco’s Minimal Essential Medium (Invitrogen, Carlsbad, CA, USA), supplemented with 10% fetal bovine serum and 5% penicillin and streptomycin. Cells were incubated in a 37 °C incubator in an atmosphere of 10% CO_2_ in air. HeLa cells were purchased from American Type Culture Collection (ATCC, Manassas, VA, USA).

### 4.3. Plasmids and AAV Vectors

The AAV vector plasmid self-complementary (sc) AAV-CBA-GFP was derived from scAAV-CBA-BGHpA (Dr. Miguel Sena-Esteves, University of Massachusetts Medical Center, Worcester, MA, USA). scAAV-rP0-hASC, scAAV-rP0-mASC and scAAV-rP0-null plasmids were derived from the plasmid scAAV-rP0-ICE. These plasmids carry two AAV inverted terminal repeat (ITR) elements, one wild type and one in which the terminal resolution site was deleted, as described [39], generating a vector that is packaged as a self-complementary (sc), double-stranded-like molecule. scAAV-rP0-null was generated by digesting the ICE transgene out of scAAV-rP0-ICE using EcoRV and then religating the blunt site. scAAV-rP0-hASC and scAAV-rP0-mASC plasmids were generated by inserting PCR-amplified hASC (600 bp) or mASC (594 bp) into scAAV-rP0-null that was linearized with EcoRV. All AAV vectors carry the bovine growth hormone polyadenylation signal. The identity of all the cloned transgenes was confirmed by sequencing. AAV1 serotype (AAV1) vectors were produced by transient co-transfection of 293T cells by calcium phosphate precipitation of vector plasmid (scAAV-rP0-ASC, scAAV-rP0-mASC, or scAAV-rP0-null), adenoviral helper plasmid pAdΔF6, and a plasmid encoding the AAV1 cap gene (pXR1), as previously described [40]. Briefly, AAV1 vectors were purified by iodixanol density gradient centrifugation. The vector containing iodixanol fraction was placed into an Amicon Ultra^®^ 100 kDa molecular weight cutoff (MWCO) 15 mL Ultracel-100 centrifugal filter (Millipore, Billerica, MA, USA) to concentrate and exchange buffer to PBS. The vector was further concentrated with Amicon Ultra^®^ 100-kDa MWCO 0.5 mL Ultracel-100 centrifugal filter (Millipore, Billerica, MA, USA) and titer (genome copies [GC]/mL) was determined by real time TaqMan PCR amplification with primers and probe specific for the bovine growth hormone polyadenylation signal. AAV1 vectors were stored at −80 °C until use.

### 4.4. Generation of Tumors and Vector Injection

Sciatic nerve schwannomas were generated by direct injection of HEI-193FC human or 08031-8FC mouse schwannoma cells into the left sciatic nerve of isoflurane-anesthetized athymic nude (nu/nu, 5–7-week-old males) or syngeneic FVB/*n* (5–7-week-old males) mice, as described [26]. Specifically, cells were implanted approximately 4 mm distal to the sciatic notch at a point midway between the sciatic notch and the trifurcation of the sciatic nerve into the common peroneal, tibial and sural branches. HEI-193FC cells or 08031-9FC were trypsinized and rinsed with cold PBS, and 30,000 cells in a volume of 0.5 µL of PBS were injected into the sciatic nerve of athymic nude mice or FVB/*n* mice, respectively, using a glass micropipette and a gas-powered microinjector (IM-300; Narishige, Tokyo, Japan). Tumor growth was monitored by in vivo bioluminescence imaging at weekly intervals, as described [28]. Briefly, mice were injected intraperitoneally (I.P.) with the Fluc substrate d-luciferin, and 10 min later, signal was acquired with a high efficiency IVIS Spectrum (Caliper Life Sciences, Hopkinton, MA, USA). Tumors were injected with AAV1-rP0-hASC, or PBS two weeks post HEI-193FC tumor-cell implantation, with vector GC in 2 µL PBS, targeting the location of the sciatic nerve where tumor cells were implanted. Tumor cell burden was tracked by in vivo bioluminescence imaging.

### 4.5. Behavioral Analysis

Behavioral testing utilized the von Frey method for pain/mechanical sensitivity, the Hargreaves plantar test for pain/thermal sensitivity, and the rotarod for gross motor function, all according to published methods [28,29]. Naïve and AAV1 immunized C57BL/6 mice were used for the behavioral experiments. All animals were allowed to habituate to the behavioral apparatus for 1 week before testing for baseline. Three baseline measurements on three separate days preceded the first injection. AAV1-rP0-mASC or AAV1-rP0-null (1 × 10^10^ gc in 2 uL PBS) were injected into the sciatic nerve of mice. Mice were then tested the day after each injection and twice per week for 8 weeks. Mechanical sensitivity of the hind paw was measured by determining withdrawal thresholds assessed with von Frey filaments employed to determine mechanical sensitivity of the plantar surface of the hind paw, as described [12]. The 50% threshold for each paw withdrawal was calculated, as previously described [41]. Thermal sensitivity of the hind paw was measured by determining withdrawal latency assessed with Hargreaves plantar test, as described [27]. A rotating rod apparatus (Columbus Instruments, Columbus, OH, USA) was used to assess motor performance. Mice were placed on the elevated accelerating rod beginning at 1 rpm/min for two trials per day twice per week. Animals’ fall latency (in seconds) was scored, as described [12].

### 4.6. Histological Analysis

After intra-sciatic injection with vector or control, animals were terminally anesthetized with isoflurane (3%) and sacrificed by neck dislocation. Sciatic nerves were removed and snap frozen for hematoxylin and eosin (H&E) as described [42]. The sciatic nerves were kept in OCT blocks at −80 °C. Sections were stained with H&E in accordance with routine protocols. For Epon staining, the sciatic nerve was removed and was fixed in glutaraldehyde. Myelin integrity of sciatic nerves injected with AAV1-rP0-mASC vector was assessed in the stained sections as previously described [12,43].

### 4.7. Generation of AAV Immunized Mice

C57BL/6 or FVB/*n* mice were injected I.P. with the 1 × 10^8^, 1 × 10^9^ or 1 × 10^10^ gc AAV1-rP0-null in PBS (total volume 200 μL). One week later, blood was collected from anesthetized mice via cardiac puncture. Blood sera were collected, as described [44] for in vitro neutralization assay and ELISA.

### 4.8. Neutralization Assay

In vitro neutralization assays were performed with mixing blood sera from the AAV1-rP0-null I.P. injected mice with AAV1-CBA-GFP. HeLa cells were seeded at 50,000 cells per well in a 24-well plate the day before the assay. Next, a dose of 5 × 10^7^ gc of AAV1-CBA-GFP was mixed with serial dilutions of mice sera. AAV1-CBA-GFP with serum from PBS injected mice served as control. These were incubated for 1 h at 37 °C before adding to cells for 1.5 h at 37 °C. After washing cells one time and replacing with complete medium, cells were incubated for 48 h before monitoring GFP expression using fluorescence microscope. Percentage of GFP positive cells was estimated via capturing six random fields of view per condition using fluorescence microscopy, and GFP-positive cells were counted using ImageJ software (*n* = 3 wells/treatment). The percent of GFP-positive cells was averaged for each microscopic field. All green cellular profiles were counted as GFP-positive cells. 

### 4.9. AAV1-ELISA Assay

High binding 96-well plate was coated with 100 uL coating buffer as control wells or 100 uL (1 × 10^10^ gc) AAV1-rP0-null virus made, covered and incubated over night at 4 °C (*n* = 3 wells/treatment). The following day, the plate wells were washed with PBS with Tween-20, 0.05% (PBST) three times then blocked with 300 uL/well blocking buffer for 1 h at room temperature. Sera were diluted 1:20 in blocking buffer and 100 uL of the diluted mix was added per well and incubated for 1.5 h at 37 °C. Secondary antibody 100 uL/well (anti-mouse IgA + IgM + IgG, 1:500 dilution or 1:250 dilution of glycerol stock) was added to each well, and incubated for 1.5 h at 37 °C. Wells were washed with PBST for 5 min on a shaker; this was repeated 6 times. Substrate luminescence was added in wash buffer 100 uL/well and read with luminometer.

### 4.10. Data Analysis

All data are presented as group averages ± standard error of the mean (SEM). All baseline behavioral values represent average of all measurements obtained before injection. Data were analyzed with GraphPad Prism and Microsoft Excel. Two-tailed *t*-test, repeated measures analysis of variance (ANOVA) and one-way ANOVA were utilized as described [45]. *p* < 0.05 was accepted as significant.

## 5. Patents

Doctors Brenner and Ahmed are inventors of submitted patent WO2018106753A1 (Methods and compositions relating the treatment of tumors).

## Data Availability

Not applicable.
